# An Improved Multioperator-Based Constrained Differential Evolution for Optimal Power Allocation in WSNs

**DOI:** 10.3390/s21186271

**Published:** 2021-09-18

**Authors:** Wei Li, Wenyin Gong

**Affiliations:** School of Computer Science, China University of Geosciences, Wuhan 430074, China; liwei2@cug.edu.cn

**Keywords:** wireless sensor networks, optimal power allocation, constrained differential evolution, multioperator adaptation, ant colony optimization

## Abstract

Optimal power allocation (OPA), which can be transformed into an optimization problem with constraints, plays a key role in wireless sensor networks (WSNs). In this paper, inspired by ant colony optimization, an improved multioperator-based constrained adaptive differential evolution (namely, IMO-CADE) is proposed for the OPA. The proposed IMO-CADE can be featured as follows: (i) to adaptively select the proper operator among different operators, the feedback of operators and the status of individuals are considered simultaneously to assign the selection probability; (ii) the constrained reward assignment is used to measure the feedback of operators; (iii) the parameter adaptation is used for the parameters of differential evolution. To extensively evaluate the performance of IMO-CADE, it is used to solve the OPA for both the independent and correlated observations with different numbers of sensor nodes. Compared with other advanced methods, simulation results clearly indicate that IMO-CADE yields the best performance on the whole. Therefore, IMO-CADE can be an efficient alternative for the OPA of WSNs, especially for WSNs with a large number of sensor nodes.

## 1. Introduction

Due to their cost-effectiveness, easy deployment, and intelligence, wireless sensor networks (WSNs) have obtained considerable attention in the last few decades [1]. Nowadays, WSN is one of the promising technologies for real-world applications [2,3]. To improve their performance and lifetime, various computational intelligence techniques have been used recently for the design of WSNs [4], such as particle swarm optimization (PSO) [5], genetic algorithms (GAs), ant colony optimization (ACO) [6], machine learning algorithms [7], lion optimization [8], negatively correlated search [9], and Fibonacci tree optimization algorithm [10].

In WSNs, there are usually many sensor nodes, which have a very limited power supply. Therefore, among various design issues, optimal power allocation (OPA) plays a key role in WSNs [11,12,13,14,15]. The OPA can be transformed into an optimization problem with constraints. Due to its nonlinear and computationally expensive (for the correlated observations) nature, the optimization of the transformed problem is difficult for traditional/analytical methods; especially, there are a large number of sensor nodes with correlated observations due to the expensive computation cost [16]. In the literature, the use of nature-inspired optimization methods for solving the OPA has received more consideration [6]. For example, in [17], PSO was used for the OPA. Boussaïd et al. [18] presented a constrained bio-geography-based optimization (BBO) and differential evolution (DE)—namely, CBBO-DE—for the OPA. In [19], combined with the penalty technique, three nature-inspired methods—cat swarm optimization (CSO), cuckoo search (CS), and PSO—were compared for solving the OPA. In [20], Tsiflikiotis et al. developed a hybrid method with teaching–learning-based optimization and Jaya algorithms. Lee compared three swarm intelligence algorithms—PSO, artificial bee colony (ABC), and continuous ACO (${\mathrm{ACO}}_{R}$)—for the OPA [21]. In [22], a constrained DE with multiple operator selection (PM-MDE) was presented, where a transformed fitness function was used for the constraints.

As mentioned above, various nature-inspired algorithms has been presented to solve the OPA in WSNs. However, there is still much room to improve the performance for the OPA by developing other advanced optimization techniques, especially for the OPA with a large number of sensor nodes and/or with the correlated observations. Based on this consideration and inspired by our previous work [22], in this paper, we propose an improved multioperator-based constrained adaptive DE, referred to as IMO-CADE, to try to effectively solve the OPA. IMO-CADE has the following three characteristics: (i) to adaptively select the proper operator among different operators, the feedback of operators and the status of individuals are considered simultaneously, such as the edge selection in ant colony optimization; (ii) the constrained reward assignment is used to measure the feedback of operators; (iii) the parameter adaptation is used for the parameters of DE. To evaluate the performance of IMO-CADE, extensive experiments have been conducted for the OPA on both the independent and correlated observations. Compared with other advanced methods, IMO-CADE can consistently provide the best results on the whole.

The rest of the paper is organized as follows: In Section 2, the background is briefly introduced, including the optimization problem formulation of the OPA, basic DE, and probability matching. The related work of nature-inspired optimization methods for the OPA are briefly reviewed in Section 3. Section 4 elaborates on the proposed IMO-CADE method. In Section 5, extensive simulations have been conducted to evaluate the performance of our proposal. Finally, Section 6 concludes the paper and points out some possible future work.

## 2. Background

In this section, first, the transformed optimization problem of the OPA is described. Then, the original DE is briefly introduced.

### 2.1. Problem Formulation

In this work, a WSN with a fusion center and *K* sensor nodes is considered. The optimization problem of the OPA can be formulated as follows (Since this paper mainly focuses on the development of efficient optimizer for the OPA, the data fusion problem formulation of the OPA in WSNs is not mentioned herein; the interested reader can refer to the work in [17] for the details.) [17,18]:

(1) $$\min_{G_{k}\geq 0}f\left(x\right)=\sum_{k=1}^{K}G_{k}^{2},$$

subject to

(2) $$\begin{array}{c} P\left(E\right)=Q\left(\frac{1}{2}\sqrt{m^{2}e^{T}A\sum {}_{K}^{-1}Ae}\right)\leq \varepsilon ,\\ G_{k}\geq 0,\\ k=1,\cdots ,K. \end{array}$$

where ε is the required fusion error probability threshold, *m* indicates the deterministic signal, $G_{k}$ is the amplifier gain at node *k*, and e is the *L*-length vector with all ones. The covariance matrix is $\sum {}_{K}=A^{T}\sum {}_{v}A+\sum {}_{w}$, where $A=diag(H_{1}G_{1},\ldots ,H_{K}G_{K})$, $\sum {}_{v}$, and $\sum {}_{w}$ are the observation and receiver noise covariances, respectively. $H_{k}$ (k=1,⋯,K) is the channel fading coefficient. In this work, two situations (i.i.d. and correlated observations) are considered.

#### 2.1.1. Independent Observations

If the local observations and the receiver noise are both i.i.d., the probability of fusion error can be simplified to

(3) $$P\left(E\right)=Q\left(\frac{m}{2}\sqrt{\sum_{k=1}^{K}\frac{H_{k}^{2}G_{k}^{2}}{\delta_{v}^{2}H_{k}^{2}G_{k}^{2}+\delta_{\omega }^{2}}}\right)\leq \varepsilon .$$

The inequality in (2) can be expressed as follows:

(4) $$\beta \leq \sqrt{\sum_{k=1}^{K}\frac{H_{k}^{2}G_{k}^{2}}{\delta_{v}^{2}H_{k}^{2}G_{k}^{2}+\delta_{\omega }^{2}}},$$

where $\beta =\frac{2}{m}Q^{-1}\left(\varepsilon \right)$ and Q(·) is the complementary Gaussian cumulative distribution function. $\delta_{v}$ means the variances of the observation noise and $\delta_{w}$ represents the receiver noise. In this work, the channel fading coefficient $H_{k}$ follows an exponential distribution (i.e., Rayleigh fading) with unit mean [18].

#### 2.1.2. Correlated Observations

If the sensor observations are spatially correlated, the observation noise covariance matrix $\sum {}_{v}$ with the correlation degree ρ can be formulated as follows:

(5) $$\sum {}_{v}=\delta_{v}^{2}\left(\begin{array}{ccccc} 1 & \rho^{d} & \cdots & \rho^{d(K-2)} & \rho^{d(K-1)}\\ \rho^{d} & 1 & \cdots & \rho^{d(K-3)} & \rho^{d(K-2)}\\ \vdots & \vdots & \ddots & \vdots & \vdots \\ \rho^{d(K-1)} & \rho^{d(K-2)} & \cdots & \rho^{d} & 1 \end{array}\right).$$

The inequality in (2) is

(6) $$\beta \leq \sqrt{e^{T}A\sum {}_{K}^{-1}Ae},$$

where $d_{j}=d\left(j-1\right),j=1,\ldots K$, which means the sensor nodes are equally spaced along a straight line. Since $\sum {}_{v}$ is not diagonal, it is difficult to evaluate $\sum {}_{K}^{-1}$ in closed form. Note that, under the correlated situation, the computational complexity for the calculation of Equation (5) is $O(K^{2})$, which means that the OPA with correlated observations is computationally expensive when *K* is large.

### 2.2. Differential Evolution

**Figure 1:**
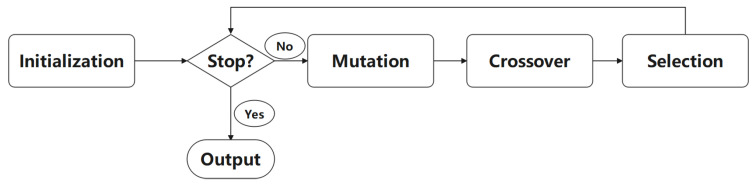
The flowchart of DE.

DE is a simple and efficient global optimizer for numerical optimization problems [23]. The flowchart of the original DE is shown in Figure 1, where there are four main procedures—i.e., population initialization, differential mutation, crossover, and selection.

#### 2.2.1. Initialization

Suppose the population contains Np solutions. For the OPA, each solution $x^{i}=\left(x_{1}^{i},\cdots ,x_{K}^{i}\right)$, i=1⋯,Np, is a *K*-dimensional real-valued vector. Initially, each variable $x_{i,k}$ is randomly generated as

(7) $$x_{k}^{i}=\mathrm{rndreal}\left(0,10\right),$$

where i=1⋯,Np and k=1,⋯,K. The lower and upper bounds of $x_{k}^{i}$ are 0 and 10, respectively. rndreal(0,10) is a real-valued random number generator from (0,10). Note that, for the OPA, $x_{k}^{i}$ represents the amplifier gain at node *k*, i.e., $G_{k}$.

#### 2.2.2. Mutation

In DE, the core operator is the *differential mutation*. The commonly used “DE/rand/1” can be formulated as

(8) $$v^{i}=x^{r1}+F\left(x^{r2}-x^{r3}\right),$$

where $v^{i}$ is generated mutant vector; r1,r2,r3∈{1,Np} satisfy r1≠r2≠r3≠i, and F∈(0,1) is a scaling factor.

#### 2.2.3. Crossover

After mutation, the crossover is performed between the target vector $x^{i}$ and the mutant vector $v^{i}$. The binomial crossover is

(9) $$u_{k}^{i}=\left\{\begin{array}{cc} v_{k}^{i}, & \mathrm{if}\;\mathrm{rndreal}\left(0,1\right)\leq Cr\;\mathrm{or}\;rand_{j}==k\\ x_{k}^{i}, & \mathrm{otherwise} \end{array}\right.,$$

where $u^{i}$ is the trial vector, $rand_{j}\in \left\{i,K\right\}$ is a randomly generated integer, and Cr∈[0,1] is the crossover rate.

#### 2.2.4. Selection

After generating the trial vector, DE performs the one-to-one tournament selection to select the solution to survive into the next generation:

(10) $$x^{i}=\left\{\begin{array}{cc} u^{i}, & \mathrm{if}\;u^{i}\;\mathrm{is}\;\mathrm{better}\;\mathrm{than}\;x^{i}\\ x^{i}, & \mathrm{otherwise} \end{array}\right..$$

The mutation, crossover, and selection will repeat until the termination criterion is met.

## 3. Related Work

As stated in [17], if the observations are i.i.d, the OPA can be derived analytically in closed form. However, if the observations are spatially correlated, the evaluation of $\sum_{K}^{-1}$ in (6) cannot be obtained analytically in closed form. Therefore, recently, various nature-inspired methods are used for the OPA.

In [17,24], PSO was employed to obtain the OPA with the constraint of a required fusion error probability threshold. To deal with the constraint, the exterior penalty function with two additional parameters (i.e., positive penalty parameter $r_{k}$ and non-negative constant *q*) is used. In [18], Boussaïd et al. hybridized BBO with DE for the OPA, where the penalty function presented in [25] was used to handle the constraint. In [19], with the dynamically modified penalty functions, CSO, CS, and PSO were empirically compared for the OPA. The results indicated that (i) when the number of the sensor nodes is small, PSO gets better results; however, (ii) CSO outperforms CS and PSO when the network consists of a large number of sensors. Tsiflikiotis et al. [20] extended their previous work [19] and presented a hybrid TLBO and Jaya (namely, TLBO-Jaya) to solve the OPA, where the proposed TLBO-Jaya was compared with TLBO, Jaya, heat transfer search algorithm, PSO, and BBO-DE. In [21], Lee used PSO, ABC, and ${\mathrm{ACO}}_{R}$ for optimizing the OPA, where Deb’s three feasibility rules [26] were used to replace the penalty function for the constraint handling. Li et al. [22] presented a constrained DE with multiple mutation strategies, where probability matching and constrained credit assignment techniques were used.

The abovementioned methods obtained promising results for solving the OPA. However, most of them are used to solve the OPA with a small number of sensor nodes, e.g., K≤50. In addition, the penalty functions usually introduce new parameters, which may influence the performance significantly. Therefore, there is still much room to develop advanced, effective, and efficient numerical methods for the OPA, especially for the OPA with a large number of sensor nodes.

## 4. Our Approach: IMO-CADE

With the main objective of finding an alternative solution for minimizing the total power consumed by the WSN, in this section, the proposed IMO-CADE is introduced in detail, including the motivations, operator pool, constrained reward assignment, improved multioperator selection technique, and parameter adaptation.

### 4.1. Motivations

As mentioned in Section 1 and Section 3, different nature-inspired methods are presented to solve the OPA. To deal with the constraints, most methods use penalty functions. However, it is difficult to set the penalty coefficients for different problems. In [21], it adopts the feasibility rules [26]. However, the feasibility rules favor the feasible solutions, which may result in trapping into the local feasible regions.

In [27], the empirical study stated that the performance of DE may significantly be improved by the mutation operators for the OPA. It is difficult to choose the most suitable mutation operators for different cases in the OPA. In our previous work [22], probability matching was used for operator selection. However, to assign the probabilities of different operators, only operator feedback is considered. Indeed, individual information should also be considered for the probability assignment.

Based on the above considerations, in this work, we propose an improved multioperator selection technique by considering both the operator feedback and individual information. In addition, to further improve the performance, parameter adaptation of DE is also used to release the setting of parameters by the users. The main techniques used in IMO-CADE are elaborated in the following subsections.

### 4.2. Operator Pool

In the DE literature, various mutation operators have been proposed [28]. In this work, to efficiently solve the OPA, two mutation operators presented in [29,30] were chosen as the operator pool—“DE/current-to-*p*best/1” with archive and “DE/rand-to-*p*best/1” with archive:

- “DE/current-to-*p*best/1” with archive:
  (11) $$v^{i}=x^{i}+F^{i}\left(x^{pbest}-x^{i}\right)+F^{i}\left(x^{r1}-{\hat{x}}^{r2}\right),$$
- “DE/rand-to-*p*best/1” with archive:
  (12) $$v^{i}=x^{r0}+F^{i}\left(x^{pbest}-x^{r0}\right)+F^{i}\left(x^{r1}-{\hat{x}}^{r2}\right),$$
  where r0,r1,r2∈{1,Np} and r0≠r1≠r2≠i. $F^{i}$ is the scaling factor of the *i*-th solution; $x^{pbest}$ is the “*p*best” solution randomly chosen from the top p% solutions of the current population; ${\hat{x}}^{r2}$ is a randomly selected solution from the union of the current population P and the archive A.

### 4.3. Boundary Constraint Handling

After generating the mutant vector with the mutation operator, some variables may be out of their boundary constraints, i.e., $v_{k}^{i}\notin \left[0,10\right]$. In this situation, the following boundary constraint handling technique (BCHT) is used:

(13) $$v_{k}^{i}=\left\{\begin{array}{cc} (0+x_{k}^{i})/2, & \mathrm{if}\;v_{k}^{i}<0\\ (10+x_{k}^{i})/2, & \mathrm{if}\;v_{k}^{i}>10\\ v_{i}^{k}, & \mathrm{otherwise} \end{array}\right..$$

### 4.4. Constrained Reward Assignment

For solving the constrained optimization problems, there are three situations of the *combined* parent population P and child population C.

(i) *Infeasible situation*: All solutions in P∪C are infeasible. Under this situation, the fitness F(x) of each solution x is its overall constraint violation (CV),
  (14) F(x)=CV(x)=P(E)−ϵ.
(ii) *Semifeasible situation*: P∪C contains both the infeasible and feasible solutions. In this situation, the solutions in the parent and child populations are combined. Then, for each solution, its objective function and CV are normalized as suggested in [31]. Afterwards, the fitness is as follows:
  (15) $$F\left(x\right)=f_{\mathrm{nor}}\left(x\right)+CV_{\mathrm{nor}}\left(x\right),$$
  where $f_{\mathrm{nor}}\left(x\right)$ and $CV_{\mathrm{nor}}\left(x\right)$ are the normalized objective function and CV, respectively. The details can be found in [31].
(iii) *Feasible situation*: all solutions in P∪C are feasible. In this situation, the fitness is the objective function:
  (16) $$F\left(x\right)=f\left(x\right)=\sum_{k=1}^{K}G_{k}^{2}.$$

According to the above fitness of each solution under situations, the relative fitness improvement is calculated as [22]

(17) $${\hat{F}}^{i}=\frac{F^{best}}{F(u^{i})}\left(F\left(x^{i}\right)-F\left(u^{i}\right)\right),$$

where i=1,⋯,Np, $F^{best}$ is the fitness of the best-so-far solution, $x^{i}$ is the target solution, and $u^{i}$ is its corresponding trial solution.

Then, based on the relative fitness improvement, the reward $R_{o}$ of the *o*-th operator is calculated by

(18) $$R_{o}=\frac{\sum {}_{i=1}^{\left|S_{o}\right|}S_{o}\left(i\right)}{\left|S_{o}\right|},$$

where $S_{o}$ is the set of all relative fitness improvements ${\hat{F}}^{i}$ of an operator $o(o=1,\cdots ,N_{o})$, $N_{o}$ is the number of operators in the pool, $\left|S_{o}\right|$ is the size of $S_{o}$, and $S_{o}\left(i\right)$ is the *i*-th relative fitness improvement of operator *o* saved in $S_{o}$.

### 4.5. Improved Multioperator Selection

In order to adaptively select a suitable operator from the pool, the selection probability of each operator is assigned. In this work, both the operator feedback and individual information are considered simultaneously.

#### 4.5.1. Probability Based on Operator Feedback

The operator feedback is measured by its constrained reward, as shown in Equation (18). Based on the reward, probability matching [32] is used to assign the probability of each operator. First, for each operator *o*, its quality is updated as

(19) $$q_{o}=q_{o}+\alpha \left(R_{o}-q_{o}\right),$$

where α∈(0,1] is the adaptation rate. Then, the probability is calculated as

(20) $$p_{o}^{\prime }=p_{\min}+\left(1-N_{o}\cdot p_{\min}\right)\frac{q_{o}}{\sum_{i=1}^{N_{o}}q_{i}},$$

where $p_{\min}\in \left(0,1\right)$ is the minimal probability of each operator.

#### 4.5.2. Probability Based on Individual Information

Generally, for different solutions in the population, different mutation operators may be more suitable. In this work, two mutation operators, i.e., “DE/current-to-*p*best/1” with archive (o=1) and “DE/rand-to-*p*best/1” with archive (o=2), are used in the pool. “DE/current-to-*p*best/1” with archive performs the local search around the target solution, and hence, is more suitable to the *better* solutions. “DE/rand-to-*p*best/1” with archive is able to provide more diversity, and hence, is more suitable to the *worse* solutions. Based on this consideration, first, the solutions in P are sorted based on the feasibility rules [26] from the best to the worst. Then, according to the individual information, the probability is assigned as

(21) $$p_{o,i}^{\prime \prime }=\left\{\begin{array}{cc} 0.9, & \mathrm{if}\;1\leq i\leq Np/2\;\&\;o==1\\ 0.1, & \mathrm{if}\;1\leq i\leq Np/2\;\&\;o==2\\ 0.1, & \mathrm{if}\;Np/2<i\leq Np\;\&\;o==2\\ 0.9, & \mathrm{if}\;Np/2<i\leq Np\;\&\;o==1, \end{array}\right.$$

where $p_{o,i}^{\prime \prime }$ is the probability for the *o*-th operator of the *i*-th solution, o=1,2, and i=1,⋯,Np.

#### 4.5.3. Final Probability Calculation

Finally, inspired by ACO [33], by combining operator feedback and individual information, the probability is calculated by

(22) $$p_{o,i}=\frac{p_{o}^{\prime }\cdot p_{o,i}^{\prime \prime }}{\sum_{j=1}^{N_{o}}p_{j}^{\prime }\cdot p_{j,i}^{\prime \prime }}.$$

### 4.6. Parameter Adaptation

To further improve the performance of IMO-CADE for the OPA (Algorithm 1), the parameter adaptation technique proposed in [29] is also used. Initially, for each target solution $x^{i}$, its associated parameters $Cr^{i}$ and $F^{i}$ are generated as

(23) $$Cr^{i}=\mathrm{Gaussian}\left(\mu_{Cr},0.1\right),$$

and

(24) $$F^{i}=\mathrm{Cauchy}\left(\mu_{F},0.1\right),$$

where “$\mathrm{Gaussian}(\mu_{Cr},0.1)$” is a Gaussian random number generator with mean $\mu_{Cr}$ and standard deviation 0.1, and “$\mathrm{Cauchy}(\mu_{F},0.1)$” is a Cauchy random number generator with location $\mu_{F}$ and scale 0.1. At the first generation, $\mu_{Cr}=0.5$ and $\mu_{F}=0.5$.
**Algorithm 1** Pseudo-code of IMO-CADE**Input:** Algorithm parameters: $Np,\mu_{Cr},\mu_{F},c,p_{\min},\alpha ,NFEs_{\max}$; WSN parameters: $K,\epsilon ,\rho ,\gamma_{0}$ **Output:** The best solution $x^{\mathrm{best}}=\left\{G_{1}^{\mathrm{best}},\cdots ,G_{K}^{\mathrm{best}}\right\}$
  1:Initialize the population P with Np solutions;  2:Evaluate the population P and set NFEs=Np;  3:For each operator, set $q_{o}=0$ and $p_{o}^{\prime }=1/2$;  4:Set A=Φ;  5:**while**$NFEs<NFEs_{\max}$**do**  6: Set $S_{Cr}=\Phi$, $S_{F}=\Phi$;  7: Sort the solutions in P based on the feasibility rules from the best to the worst;  8: For each solution in P, set $p_{o,i}^{\prime \prime }$ according to Equation (21);  9: For each solution, calculate $p_{o,i}$ based on Equation (22);10: **for** i=1 to Np **do**11:  Select the operator $o^{i}$ based on the selection probability;12:  Calculate $Cr^{i}$ and $F^{i}$ based on Equations (23) and (24), respectively;13:  Generate $u^{i}$ and deal with the violated variables based on Equation (13);14:  Evaluate $u^{i}$, NFEs=NFEs+1, and $C\leftarrow u^{i}$;15:** end for**16: M←P∪C;17: For each solution x in M, calculate F(x) under the current situation;18: **for** i=1 to Np **do**19:  **if** $F\left(u^{i}\right)\leq F\left(x^{i}\right)$ **then**20:   $A\leftarrow x^{i}$;21:   Calculate ${\hat{F}}^{i}$ based on Equation (17) and save it into $S_{o}\left(i\right)$;22:   $S_{Cr}\leftarrow Cr^{i}$ and $S_{F}\leftarrow F^{i}$;23:   Replace $x^{i}$ with $u^{i}$;24:  **else**25:   Set ${\hat{F}}^{i}=0$ and save it into $S_{o}\left(i\right)$;26:  **end if**27: **end for**28: Update $\mu_{Cr}$ and $\mu_{F}$;29: **for** o=1 to 2 **do**30:  Calculate $R_{o}$ based on Equation (18);31:  Update $p_{o}^{\prime }$ based on Equation (20);32: **end for**33:**end while**

After that, if the trial solution $u^{i}$ is better than its target solution $x^{i}$, the parameters $Cr_{i}$ and $F_{i}$ are saved in $S_{Cr}$ and $S_{F}$, respectively. Subsequently, $\mu_{Cr}$ and $\mu_{F}$ are updated as follows:

(25) $$\mu_{Cr}=\left(1-c\right)\cdot \mu_{Cr}+c\cdot {\mathrm{mean}}_{A}\left(S_{Cr}\right),$$

and

(26) $$\mu_{F}=\left(1-c\right)\cdot \mu_{F}+c\cdot {\mathrm{mean}}_{L}\left(S_{F}\right),$$

where c=0.1 is a parameter, ${\mathrm{mean}}_{A}\left(S_{Cr}\right)$ calculates the arithmetic mean of $S_{Cr}$, and ${\mathrm{mean}}_{L}\left(S_{F}\right)$ calculates the Lehmer mean of $S_{F}$ [29].

### 4.7. Framework of IMO-CADE

By integrating the abovementioned techniques, the pseudo-code of IMO-CADE is shown in Algorithm 1, where NFEs is the current number of fitness evaluations and $NFEs_{\max}$ is the maximal NFEs. First, the population P is initialized randomly; then, each solution is evaluated. The initial quality of each operator is set to $q_{o}=0$ and $p_{o}^{\prime }=1/2$. The archive A is set to be empty. In the main loop, it works as follows:

- At each generation, $S_{Cr}$ and $S_{F}$ are set to be empty.
- In lines 8–9, the selection probability $p_{o,i}$ is calculated.
- In line 11, for each solution, one operator $o^{i}$ is selected based on the selection probability and roulette wheel selection.
- In lines 12–14, the trial solution is generated according to the selected operator and the generated parameters. The violated variables are handled based on the BCHT.
- In lines 16–17, the parent population P and the child population C are combined. Then, the feasibility situation is checked, followed by the transformed fitness calculation by Equations (14)–(16).
- In line 19, the trial solution $u^{i}$ is compared with its target solution $x^{i}$ based on the transformed fitness.
- If $u^{i}$ is better than $x^{i}$, in lines 20–22, the worse $x^{i}$ is saved into archive A. Note that, when |A|>Np, |A|−Np solutions are randomly removed from A to keep |A|=Np. The relative fitness improvement and the successful parameters $Cr^{i},F^{i}$ are saved.
- In line 23, $x^{i}$ is replaced by $u^{i}$ for the next generation.
- In lines 28–32, $\mu_{Cr},\mu_{F},R_{o}$, and $p_{o}^{\prime }$ are updated accordingly.

#### Remarks

It is worth noting that IMO-CADE is an improved version of our previous work (PM-MDE) in [22]. However, there are several important differences between IMO-CADE and PM-MDE:

(1) The core difference is that, in IMO-CADE, both the operator feedback and individual information are considered together to update the operator selection probability, whereas in PM-MDE, only the operator feedback is used.
(2) In IMO-CADE, a new BCHT is developed for the OPA.
(3) The operators used in the operator pool are different between IMO-CADE and PM-MDE.

## 5. Results and Analysis

In this section, the proposed IMO-CADE is used to solve the OPA with different sensor nodes under independent or correlated observations.

### 5.1. Parameter Settings

For IMO-CADE, the following parameter settings are used:

- Np=100;
- $\mu_{Cr}=0.5$, $\mu_{F}=0.5$, and c=0.1;
- $p_{\min}=0.05$ and α=0.3;
- $NFEs_{\max}=3000$;
- Number of independent runs = 30;
- Observation signal-to-noise ratio (SNR), $\gamma_{0}=10$ dB.

In this work, the WSN consists of a fusion center and many spatially separated sensors, which perform amplify-and-forward local processing of their observations independently. The OPA problem was considered for the decision fusion of a deterministic signal in an inhomogeneous WSN. The channel fading coefficients follow an exponential distribution (i.e., Rayleigh fading) with unit mean. All algorithms were implemented in C++. The simulations were executed on a desktop PC with an Intel Xeon E5-2620 processor @ 2.40 GHz, 32 GB RAM, using the Windows 10 64-bit OS.

### 5.2. Comparison with Other Advanced DEs

To evaluate the performance of IMO-CADE, it is compared with five other DE variants for the OPA with different sensor nodes under i.i.d. observations. The five DE methods are as follows: (i) SaDE [34], (ii) JADE [29], (iii) OrSHADE [35], (iv) COLSHADE [36], and (v) CADE. The first four DE methods obtained promising results in the literature (To make a fair comparison, the parameters of the four DE variants are set as the same as those used in the original literature. All methods use $NFEs_{\max}=3000$), for example, COLSHADE is one of the winners in the CEC-2020 competition for the real-world constrained single-objective optimization. CADE is a variant of IMO-CADE. The only difference between IMO-CADE and CADE is that only the operator feedback is used for operator selection in CADE.

**Figure 2:**
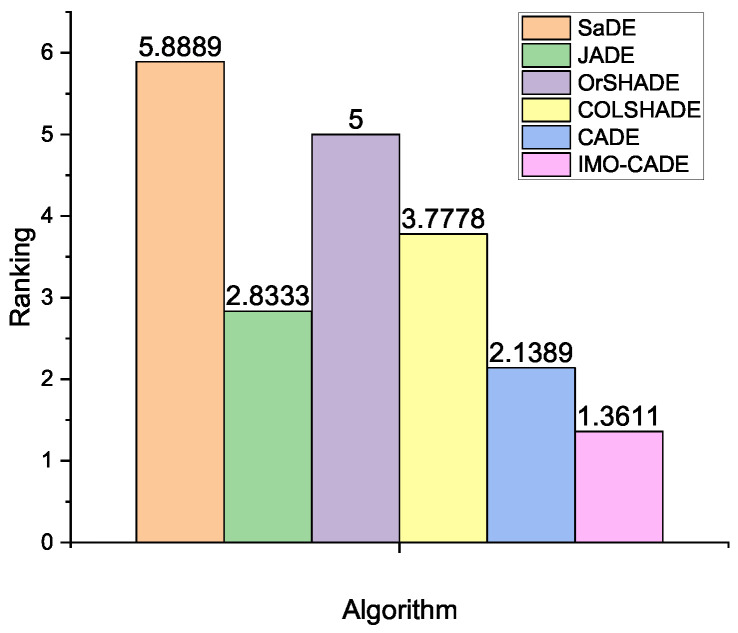
Average rankings of different DE variants obtained by the Friedman test for the OPA with i.i.d. observations, where the *p*-value computed by Iman and Daveport test is 0.

The detailed results are reported in Table 1 for K=10,20,50,100,150,200, and ϵ=0.1,0.01,0.001, where the “mean ± standard deviation” value is provided for each method on each case. All results are averaged over 30 runs. The best mean values are highlighted in **boldface**. Based on the Wilcoxon test at α=0.05 by the KEEL software [37], in the last row of Table 1, “w/t/l” indicates that IMO-CADE performs significantly better than, similar to, or significantly worse than its competitors in *w*, *t*, or *l* cases, respectively. Moreover, according to the multiple-problem analysis by the Friedman test [37], the averaging rankings of the six DE variants are plotted in Figure 2. The multiple-problem analysis by the Wilcoxon test for IMO-CADE vs. other DEs is given in Table 2.

From Table 1 and Table 2, and Figure 2, the following can be observed:

- IMO-CADE can obtain the best ranking based on the Friedman test, followed by CADE and JADE. In addition, the *p*-value computed by Iman and Daveport test is 0, which means that the performance of the six compared DEs are significantly different based on the multiple-problem analysis.
- According to the Wilcoxon test, IMO-CADE significantly outperforms SaDE, JADE, OrSHADE, COLSHADE, and CADE on 16, 12, 16, 16, and 11 cases, respectively. Compared with JADE and CADE, IMO-CADE obtains similar results on 4 and 5 cases, respectively. IMO-CADE is not outperformed by other DEs in any case.
- IMO-CADE obtains similar mean values compared with JADE and CADE when K≤20. However, when K≥50, IMO-CADE can consistently provide the best results in all cases.
- Compared with CADE, IMO-CADE obtains better results. This means that combining the operator feedback with the individual information for operator selection is really effective to improve the performance of CADE for the OPA.
- The results in Table 2 clearly show that IMO-CADE obtains significantly better results than other DEs based on multiple-problem analysis by the Wilcoxon test.

Therefore, based on the above analysis, it is clear that IMO-CADE yields the best results than other compared DE variants on the whole, especially for the OPA with a large number of sensor nodes.

### 5.3. Comparison with Reported Results

In this section, the results of IMO-CADE are compared with the reported results of other methods, i.e., CBBO [18], CDE [18], CBBO-DE [18], and PM-MDE [22], under both i.i.d. and correlated observations. Note that the five compared methods consume the same $NFEs_{\max}=3000$ for fair comparison.

#### 5.3.1. Under i.i.d. Observations

**Figure 3:**
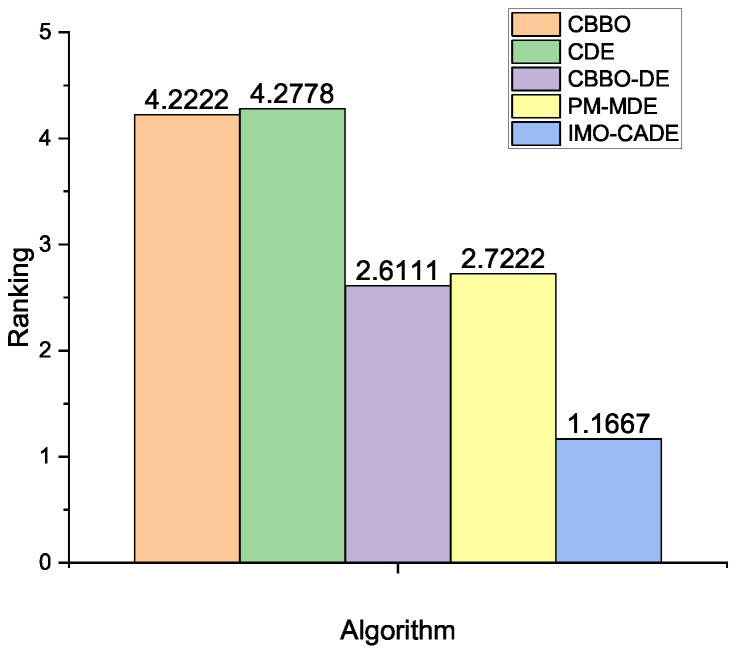
Average rankings of different methods obtained by the Friedman test for the OPA with the reported results under i.i.d. observations, where the *p*-value computed by Iman and Daveport test is 2.15 × $10^{-7}$.

The results of the five compared methods for K=10, 20, 50, and ϵ=0.1, 0.01, 0.001 are shown in Table 3. In addition, the results of the multiple-problem analysis by the Wilcoxon and Friedman tests (To calculate the statistical results, “NF” is set to be 1000.) are provided in Table 4 and Figure 3, respectively.

From Table 3, we can see that IMO-CADE can consistently obtain the best results compared with the other four methods. Additionally, the results in Table 4 show that our approach can perform significantly better than CBBO, CDE, and PM-MDE. Figure 3 confirms that IMO-CADE obtains the best ranking, followed by CBBO-DE and PM-MDE.

#### 5.3.2. Under Correlated Observations

**Figure 4:**
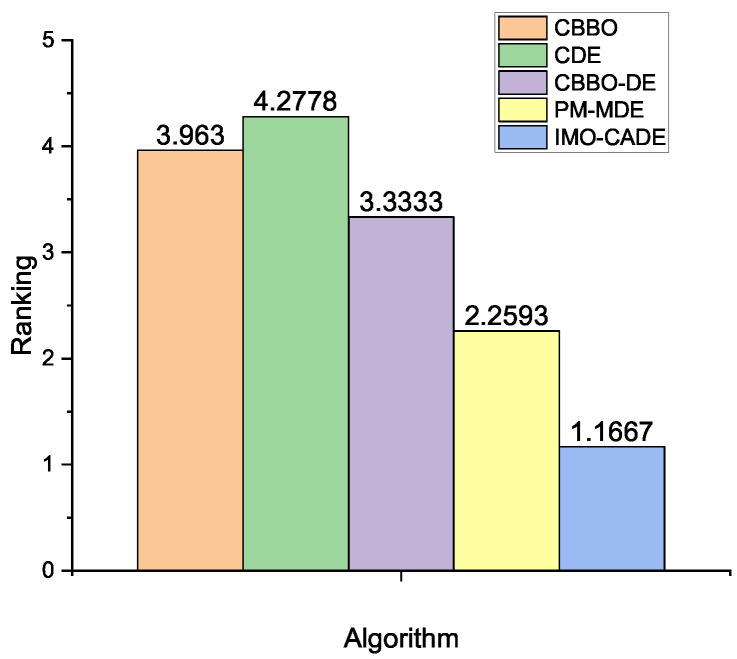
Average rankings of different methods obtained by the Friedman test for the OPA with the reported results under correlated observations, where the *p*-value computed by Iman and Daveport test is 0.

As mentioned in Section 2.1.2, when the observations are correlated, i.e., ρ≠0, the optimization of OPA is more complicated and computationally expensive. In this section, IMO-CADE is compared with CBBO, CDE, CBBO-DE, and PM-MDE with K=10, 20, 50, ρ=0.01, 0.1, 0.5, and ϵ=0.1, 0.01, 0.001. The detailed results are reported in Table 5. Moreover, the multiple-problem statistical results based on the Wilcoxon and Friedman tests are provided in Table 6 and Figure 4, respectively.

The results in Table 4 and Table 5, and Figure 4 again confirms that IMO-CADE can get the best overall results in all cases. It is able to significantly outperform CBBO, CDE, CBBO-DE, and PM-MDE based on the Wilcoxon test. Further, it obtains the best ranking based on the Friedman test.

Based on the comparison with the reported results of other methods under both i.i.d. and correlated observations with different situations (i.e., number of sensor nodes, fusion errors, and correlation degrees), the results clearly show that the proposed IMO-CADE can provide significantly better results than the compared methods. Therefore, IMO-CADE can be an effective and efficient alternative to solve the OPA of WSNs.

### 5.4. Discussions

In previous subsections, the superiority of IMO-CADE is verified on both the i.i.d. and correlated observations with different numbers of sensors. Herein, the gain allocated to each sensor of different methods is discussed. Table 7 reports the results comparison between analytical and different numerical methods with K=10, $\gamma_{0}=10$ dB, and ρ=0. Table 8 reports the results of different numerical methods with K=10, $\gamma_{0}=10$ dB, and ρ=0.1. The best results of f(x) are highlighted in boldface. In the two tables, IMO-CADE is compared with the reported results of CBBO, CDE, and CBBO-DE in [18].

From the results in Table 7 and Table 8, the following can be seen:

- Based on the gain allocated to each sensor, the sensors with poor channels can be turned off to save system power. Based on the gain allocated to each node, we can decide that the sensors with good channel fading coefficients are assigned to more power; on the other hand, sensors with poor channels are allocated less power.
- For the i.i.d. observations, the numerical results of CDE, CBBO-DE, and IMO-CADE closely match with the analytical results.
- When the observations are correlated, the sensors need more power compared with the i.i.d. observations.
- For both i.i.d. and correlated observations, IMO-CADE provides the best results of f(x) compared with other methods.

## 6. Conclusions

The OPA is one of key issues in developing WSNs. Hence, the design of effective optimization techniques for the OPA is important and obtains more attention. Based on this consideration, in this paper, we propose the IMO-CADE for the OPA. To effectively solve the OPA, in IMO-CADE, both operator feedback and individual information are considered simultaneously for multioperator selection. In addition, the parameter adaptation, efficient BCHT, and constrained reward assignment techniques are developed. IMO-CADE is extensively compared with other methods under various situations. The results clearly indicate the following:

- The proposed modifications in IMO-CADE can improve its performance for the OPA under different situations.
- With respect to the performance of the overall system power f(x), IMO-CADE is superior to other methods in all cases, especially for the WSN with a large number of sensor nodes.
- Considering gain allocation, the numerical results of IMO-CADE agree well with the analytical results.
- IMO-CADE can be an effective alternative for the OPA and other complex optimization problems of WSNs.

When the observations of sensor nodes are correlated, the OPA is computationally expensive—especially if there are a large number of sensor nodes. In the near future, combining IMO-CADE with surrogate models [38] is a promising way to efficiently solve the OPA under correlated observations. In addition, due to the similarity between the OPA and the lighting in long tunnels [39], IMO-CADE will be used for the lighting problem in long tunnels in future work. In this work, the simulations are performed to evaluate the performance of IMO-CADE. In future work, another interesting direction is using IMO-CADE in real networks.

The source code of IMO-CADE can be obtained from Dr. Gong upon request.

## Figures and Tables

**Table 1:** Comparison of different DE variants for the OPA with different sensor nodes under i.i.d. observations.

*K*	ϵ	SaDE	JADE	OrSHADE	COLSHADE	CADE	IMO-CADE
10	0.1	3.1762 ± 1.45 × $10^{-3}$	3.1723 ± 2.41 × $10^{-5}$	3.1742 ± 6.00 × $10^{-4}$	3.1732 ± 4.30 × $10^{-4}$	3.1723 ± 1.15 × $10^{-5}$	3.1723 ± 1.93 × $10^{-5}$
	0.01	15.1315 ± 7.00 × $10^{-4}$	15.1303 ± 7.19 × $10^{-11}$	15.1314 ± 6.51 × $10^{-4}$	15.1304 ± 1.30 × $10^{-4}$	15.1303 ± 5.69 × $10^{-12}$	15.1303 ± 5.55 × $10^{-13}$
	0.001	40.2599 ± 6.18 × $10^{-3}$	40.2400 ± 1.82 × $10^{-4}$	40.2469 ± 2.63 × $10^{-3}$	40.2430 ± 2.02 × $10^{-3}$	40.2400 ± 1.57 × $10^{-4}$	40.2400 ± 1.94 × $10^{-4}$
20	0.1	1.9541 ± 5.47 × $10^{-3}$	1.9317 ± 8.25 × $10^{-5}$	1.9379 ± 1.34 × $10^{-3}$	1.9365 ± 1.21 × $10^{-3}$	1.9317 ± 2.11 × $10^{-5}$	1.9317 ± 7.55 × $10^{-5}$
	0.01	9.1215 ± 9.12 × $10^{-3}$	9.0970 ± 6.50 × $10^{-6}$	9.1135 ± 4.04 × $10^{-3}$	9.1111 ± 5.12 × $10^{-3}$	9.0970 ± 9.79 × $10^{-6}$	9.0970 ± 9.67 × $10^{-6}$
	0.001	21.6245 ± 1.28 × $10^{-2}$	21.5961 ± 9.98 × $10^{-5}$	21.6252 ± 6.77 × $10^{-3}$	21.6286 ± 1.08 × $10^{-2}$	21.5962 ± 2.27 × $10^{-4}$	21.5962 ± 2.03 × $10^{-4}$
50	0.1	1.2003 ± 4.88 × $10^{-2}$	0.8659 ± 3.35 × $10^{-4}$	0.8804 ± 2.42 × $10^{-3}$	0.8766 ± 1.87 × $10^{-3}$	0.8660 ± 3.39 × $10^{-4}$	0.8656 ± 7.95 × $10^{-5}$
	0.01	4.8931 ± 8.78 × $10^{-2}$	4.3288 ± 4.22 × $10^{-3}$	4.3873 ± 9.55 × $10^{-3}$	4.3834 ± 8.23 × $10^{-3}$	4.3300 ± 6.23 × $10^{-3}$	4.3254 ± 3.90 × $10^{-3}$
	0.001	10.6059 ± 1.11 × $10^{-1}$	9.8982 ± 9.14 × $10^{-3}$	10.0035 ± 1.62 × $10^{-2}$	10.0007 ± 1.22 × $10^{-2}$	9.8955 ± 1.05 × $10^{-2}$	9.8904 ± 6.81 × $10^{-3}$
100	0.1	11.8999 ± 1.52 × $10^{0}$	0.8762 ± 1.77 × $10^{-2}$	0.9472 ± 2.06 × $10^{-2}$	0.9032 ± 1.14 × $10^{-2}$	0.8656 ± 1.06 × $10^{-2}$	0.8539 ± 1.25 × $10^{-2}$
	0.01	12.1699 ± 1.30 × $10^{0}$	3.9931 ± 5.92 × $10^{-2}$	4.1652 ± 6.26 × $10^{-2}$	4.0969 ± 3.84 × $10^{-2}$	3.9818 ± 3.97 × $10^{-2}$	3.9329 ± 2.35 × $10^{-2}$
	0.001	16.6726 ± 6.26 × $10^{-1}$	8.6754 ± 6.95 × $10^{-2}$	8.9279 ± 6.85 × $10^{-2}$	8.8313 ± 4.71 × $10^{-2}$	8.6435 ± 4.76 × $10^{-2}$	8.5999 ± 5.39 × $10^{-2}$
150	0.1	79.5028 ± 9.10 × $10^{0}$	1.2345 ± 9.28 × $10^{-2}$	1.4707 ± 1.18 × $10^{-1}$	1.1482 ± 3.41 × $10^{-2}$	1.1142 ± 5.19 × $10^{-2}$	1.0252 ± 4.80 × $10^{-2}$
	0.01	85.3694 ± 1.02 × $10^{1}$	4.6995 ± 1.14 × $10^{-1}$	5.0791 ± 1.45 × $10^{-1}$	4.6995 ± 5.62 × $10^{-2}$	4.5959 ± 1.02 × $10^{-1}$	4.4696 ± 1.10 × $10^{-1}$
	0.001	80.4239 ± 1.54 × $10^{1}$	9.6689 ± 1.51 × $10^{-1}$	10.2374 ± 2.06 × $10^{-1}$	9.7378 ± 9.40 × $10^{-2}$	9.5034 ± 1.54 × $10^{-1}$	9.3211 ± 1.18 × $10^{-1}$
200	0.1	224.0768 ± 1.57 × $10^{1}$	2.0922 ± 2.20 × $10^{-1}$	3.0493 ± 4.45 × $10^{-1}$	1.6664 ± 1.24 × $10^{-1}$	1.5992 ± 1.62 × $10^{-1}$	1.3095 ± 1.31 × $10^{-1}$
	0.01	239.8870 ± 1.63 × $10^{1}$	4.8360 ± 3.22 × $10^{-1}$	5.8158 ± 4.71 × $10^{-1}$	4.3757 ± 1.90 × $10^{-1}$	4.1938 ± 2.22 × $10^{-1}$	3.8791 ± 1.46 × $10^{-1}$
	0.001	237.0817 ± 2.03 × $10^{1}$	8.5756 ± 3.73 × $10^{-1}$	9.5102 ± 5.91 × $10^{-1}$	8.0990 ± 2.37 × $10^{-1}$	7.8208 ± 2.74 × $10^{-1}$	7.4426 ± 2.80 × $10^{-1}$
w/t/l		16/0/0	12/4/0	16/0/0	16/0/0	11/5/0	-

**Table 2:** Results obtained by the Wilcoxon test for IMO-CADE vs. other DEs under i.i.d. observations. Hereinafter, p≤0.05 indicates that IMO-CADE significantly outperforms its competitors overall.

IMO-CADE vs.	$R^{+}$	$R^{-}$	*p*-Value
SaDE	171.0	0.0	7.63 × $10^{-6}$
JADE	160.5	10.5	3.74 × $10^{-4}$
OrSHADE	171.0	0.0	7.63 × $10^{-6}$
COLSHADE	162.0	9.0	1.53 × $10^{-5}$
CADE	160.5	10.5	3.74 × $10^{-4}$

**Table 3:** Comparison with the reported results for the OPA under i.i.d. observations, where “NF” indicates that no feasible solution is obtained.

*K*	ϵ	CBBO		CDE		CBBO-DE		PM-MDE		IMO-CADE	
10	0.1	3.1991	1.64 × $10^{-2}$	3.1732	5.83 × $10^{-4}$	3.1725	1.18 × $10^{-6}$	3.1727	9.28 × $10^{-5}$	3.1723	1.93 × $10^{-5}$
	0.01	15.1680	3.31 × $10^{-4}$	15.1310	2.77 × $10^{-8}$	11.5300	8.07 × $10^{-10}$	15.1300	3.54 × $10^{-8}$	15.1303	5.55 × $10^{-13}$
	0.001	NF	NF	40.2450	5.59 × $10^{-6}$	40.2450	5.59 × $10^{-6}$	40.3170	3.95 × $10^{-4}$	40.2400	1.94 × $10^{-4}$
20	0.1	2.0406	3.91 × $10^{-2}$	2.0485	1.25 × $10^{-1}$	1.9333	1.49 × $10^{-3}$	1.9343	1.38 × $10^{-3}$	1.9317	7.55 × $10^{-5}$
	0.01	9.2443	7.93 × $10^{-2}$	9.1201	1.51 × $10^{-2}$	9.0985	6.79 × $10^{-4}$	9.1009	4.03 × $10^{-3}$	9.0970	9.67 × $10^{-6}$
	0.001	21.7400	6.07 × $10^{-2}$	21.6260	2.98 × $10^{-2}$	21.5980	6.42 × $10^{-4}$	21.6010	4.30 × $10^{-3}$	21.5962	2.03 × $10^{-4}$
50	0.1	1.4513	1.57 × $10^{-1}$	3.7790	4.12 × $10^{-1}$	2.7516	3.91 × $10^{-1}$	1.1192	6.22 × $10^{-2}$	0.8656	7.95 × $10^{-5}$
	0.01	4.9231	1.48 × $10^{-1}$	6.8290	3.59 × $10^{-1}$	6.2178	5.08 × $10^{-1}$	4.7101	8.96 × $10^{-2}$	4.3254	3.90 × $10^{-3}$
	0.001	10.4960	1.49 × $10^{-1}$	14.3240	1.49 × $10^{0}$	11.0930	6.01 × $10^{-1}$	10.3060	1.20 × $10^{-1}$	9.8904	6.81 × $10^{-3}$

**Table 4:** Results obtained by the Wilcoxon test for IMO-CADE vs. other methods with reported results under i.i.d. observations.

IMO-CADE vs.	$R^{+}$	$R^{-}$	*p*-Value
CBBO	45.0	0.0	3.91 × $10^{-3}$
CDE	45.0	0.0	3.91 × $10^{-3}$
CBBO-DE	36.0	9.0	1.29 × $10^{-1}$
PM-MDE	40.5	4.5	7.81 × $10^{-3}$

**Table 5:** Comparison with the reported results for the OPA under correlated observations.

K=10											
ρ	ϵ	**CBBO**		**CDE**		**CBBO-DE**		**PM-MDE**		**IMO-CADE**	
0.01	0.1	3.2129	2.17 × $10^{-2}$	3.1847	6.74 × $10^{-4}$	3.1833	3.86 × $10^{-4}$	3.1834	1.60 × $10^{-4}$	3.1830	9.53 × $10^{-6}$
	0.01	15.3070	7.97 × $10^{-4}$	15.2560	2.39 × $10^{-8}$	15.2550	1.89 × $10^{-9}$	15.2550	3.34 × $10^{-8}$	15.2547	6.89 × $10^{-13}$
	0.001	NF	NF	40.9860	3.00 × $10^{-6}$	41.0460	4.63 × $10^{-4}$	40.9800	3.45 × $10^{-4}$	40.9795	2.94 × $10^{-4}$
0.1	0.1	3.3100	1.77 × $10^{-2}$	3.2900	8.43 × $10^{-4}$	3.2800	1.12 × $10^{-4}$	3.2792	9.96 × $10^{-5}$	3.2789	4.19 × $10^{-5}$
	0.01	16.6000	4.30 × $10^{-4}$	16.6000	2.21 × $10^{-7}$	16.6000	3.56 × $10^{-9}$	16.4890	4.61 × $10^{-6}$	16.4885	1.05 × $10^{-9}$
	0.001	NF	NF	48.9850	1.49 × $10^{-7}$	49.0770	7.81 × $10^{-4}$	48.6440	1.12 × $10^{-10}$	48.6440	3.38 × $10^{-13}$
0.5	0.1	3.8800	1.87 × $10^{-2}$	3.8600	1.39 × $10^{-3}$	3.8600	2.32 × $10^{-4}$	3.5839	8.56 × $10^{-4}$	3.5824	5.77 × $10^{-5}$
	0.01	3.5100	6.13 × $10^{-3}$	34.4000	1.85 × $10^{-6}$	34.3000	8.08 × $10^{-7}$	22.8030	8.88 × $10^{-4}$	22.8014	9.16 × $10^{-6}$
	0.001	NF	NF	734.2400	1.88 × $10^{-4}$	735.1400	6.63 × $10^{-2}$	107.7800	6.79 × $10^{0}$	105.5153	6.72 × $10^{-13}$
K=20											
ρ	ϵ	**CBBO**		**CDE**		**CBBO-DE**		**PM-MDE**		**IMO-CADE**	
0.01	0.1	2.0292	4.15 × $10^{-2}$	2.0127	1.51 × $10^{-2}$	1.9396	2.14 × $10^{-3}$	1.9394	1.79 × $10^{-3}$	1.9373	1.73 × $10^{-5}$
	0.01	9.3053	7.68 × $10^{-2}$	91.7670	1.03 × $10^{-2}$	9.1607	8.98 × $10^{-4}$	91.6340	3.74 × $10^{-3}$	9.1588	2.00 × $10^{-6}$
	0.001	2.1980	4.97 × $10^{-2}$	21.8600	1.92 × $10^{-2}$	21.8420	3.99 × $10^{-3}$	21.8420	3.14 × $10^{-3}$	21.8383	8.06 × $10^{-6}$
0.1	0.1	2.0913	4.71 × $10^{-2}$	2.0799	1.29 × $10^{-1}$	1.9905	1.32 × $10^{-3}$	1.9908	3.58 × $10^{-3}$	1.9871	9.65 × $10^{-6}$
	0.01	99.2370	5.59 × $10^{-2}$	9.8126	3.08 × $10^{-2}$	9.7894	9.19 × $10^{-4}$	97.5540	5.49 × $10^{-3}$	9.7484	9.59 × $10^{-5}$
	0.001	24.5070	8.38 × $10^{-2}$	24.3400	1.49 × $10^{-2}$	24.3240	1.96 × $10^{-3}$	24.1820	3.48 × $10^{-3}$	24.1772	6.46 × $10^{-6}$
0.5	0.1	2.3633	2.55 × $10^{-2}$	2.3406	3.53 × $10^{-2}$	2.3026	2.19 × $10^{-3}$	2.1879	7.40 × $10^{-3}$	2.1780	1.43 × $10^{-3}$
	0.01	15.9850	5.07 × $10^{-2}$	15.8750	7.75 × $10^{-3}$	15.8650	3.27 × $10^{-3}$	12.5470	8.55 × $10^{-2}$	12.4527	1.34 × $10^{-3}$
	0.001	63.8450	9.39 × $10^{-1}$	60.9090	2.30 × $10^{0}$	60.6850	6.51 × $10^{-2}$	36.2470	9.15 × $10^{-2}$	36.1418	1.26 × $10^{-2}$
K=50											
ρ	ϵ	**CBBO**		**CDE**		**CBBO-DE**		**PM-MDE**		**IMO-CADE**	
0.01	0.1	1.5072	1.35 × $10^{-1}$	3.5357	4.37 × $10^{-1}$	2.7742	5.61 × $10^{-1}$	1.2346	1.11 × $10^{-1}$	0.8687	2.08 × $10^{-4}$
	0.01	4.9139	1.36 × $10^{-1}$	6.8366	2.26 × $10^{-1}$	6.0532	7.98 × $10^{-1}$	4.8249	1.31 × $10^{-1}$	4.3582	2.40 × $10^{-3}$
	0.001	10.6430	2.23 × $10^{-1}$	12.0540	2.28 × $10^{-1}$	10.7460	3.31 × $10^{-1}$	10.5210	7.40 × $10^{-2}$	9.9713	2.49 × $10^{-3}$
0.1	0.1	1.4947	1.64 × $10^{-1}$	3.7939	3.94 × $10^{-1}$	3.0813	4.30 × $10^{-1}$	1.2406	9.45 × $10^{-2}$	0.8960	4.22 × $10^{-5}$
	0.01	5.2413	1.67 × $10^{-1}$	7.0517	2.90 × $10^{-1}$	6.6532	1.01 × $10^{0}$	5.1088	8.14 × $10^{-2}$	4.6572	5.00 × $10^{-3}$
	0.001	11.3850	1.54 × $10^{-1}$	13.0250	2.32 × $10^{-1}$	11.6690	5.81 × $10^{-1}$	11.2840	1.20 × $10^{-1}$	10.7169	2.02 × $10^{-3}$
0.5	0.1	1.6223	1.80 × $10^{-1}$	4.1081	6.54 × $10^{-1}$	2.9672	4.70 × $10^{-1}$	1.3432	8.87 × $10^{-2}$	1.0055	2.86 × $10^{-3}$
	0.01	7.1915	1.50 × $10^{-1}$	8.5854	1.20 × $10^{-1}$	7.4210	3.24 × $10^{-1}$	6.2096	1.13 × $10^{-1}$	5.6704	5.32 × $10^{-2}$
	0.001	18.8410	1.20 × $10^{-1}$	19.3060	4.42 × $10^{-1}$	18.4490	3.68 × $10^{-2}$	14.8240	1.54 × $10^{-1}$	13.8922	4.27 × $10^{-2}$

**Table 6:** Results obtained by the Wilcoxon test for IMO-CADE vs. other methods with reported results under correlated observations.

IMO-CADE vs.	$R^{+}$	$R^{-}$	*p*-Value
CBBO	335.0	43.0	1.84 × $10^{-4}$
CDE	378.0	0.0	1.49 × $10^{-8}$
CBBO-DE	364.5	13.5	2.98 × $10^{-8}$
PM-MDE	373.0	5.0	1.49 × $10^{-7}$

**Table 7:** Comparison between analytical and different numerical methods to gain allocation with K=10, $\gamma_{0}=10$ dB, and ρ=0.

Sensor	ϵ=0.1					ϵ=0.01				
	Analytical	CBBO	CDE	CBBO-DE	IMO-CADE	Analytical	CBBO	CDE	CBBO-DE	IMO-CADE
G1	1.0362	1.0823	1.0392	1.0353	1.0361	1.6172	1.5500	1.5894	1.5925	1.5926
G2	0.9972	0.9838	0.9985	0.9977	0.9972	1.5888	1.6000	1.5826	1.5821	1.5821
G3	0.8834	0.8619	0.8853	0.8826	0.8836	1.5555	1.5185	1.5469	1.5496	1.5483
G4	0.4823	0.5219	0.4623	0.4812	0.4825	1.4666	1.4610	1.4423	1.4381	1.4379
G5	0.3021	0.1330	0.3061	0.3066	0.3010	1.4616	1.4231	1.4069	1.4050	1.4050
G6	0	0.0031	0.0655	0.0135	0.0111	1.4107	1.3738	1.3600	1.3606	1.3605
G7	0	0.0656	0.0117	0.0142	0.0029	1.1231	1.3503	1.3405	1.3404	1.3420
G8	0	0.0067	0.0006	0.0033	1.14 × $10^{-4}$	0	0.0056	0.0070	0.0053	1.20 × $10^{-6}$
G9	0	0.0020	0.0035	0.0004	8.97 × $10^{-5}$	0	0.0173	0.0037	0.0058	1.78 × $10^{-7}$
G10	0	0.0076	0.0010	0.0046	4.28 × $10^{-5}$	0	0.0016	0.0032	0.0013	1.10 × $10^{-7}$
f(x)	3.1723	3.1766	3.1725	3.1723	3.1723	15.09782	15.13894	15.13036	15.13032	15.13027

**Table 8:** Comparison of different numerical methods to gain allocation with K=10, $\gamma_{0}=10$ dB, and ρ=0.1.

Sensor	ϵ=0.1				ϵ=0.01			
	CBBO	CDE	CBBO-DE	IMO-CADE	CBBO	CDE	CBBO-DE	IMO-CADE
G1	1.0942	1.0507	1.0475	1.0442	1.6645	1.6733	1.6751	1.6717
G2	0.8958	0.9567	0.9606	0.9574	1.5635	1.5863	1.5869	1.5754
G3	0.8978	0.8850	0.8784	0.8818	1.5626	1.5775	1.5771	1.5751
G4	0.5006	0.5091	0.5015	0.5103	1.5491	1.4767	1.4805	1.5086
G5	0.4439	0.4246	0.4431	0.4508	1.5218	1.4797	1.4809	1.4817
G6	0.1227	0.1997	0.1959	0.1471	1.4327	1.4504	1.4429	1.4812
G7	0.1421	0.0371	0.0743	0.0952	1.4659	1.5110	1.5111	1.4374
G8	0.0263	0.0197	0.0067	0.0012	0.0019	0.0245	0.0130	3.84 × $10^{-5}$
G9	0.0304	0.0141	0.0022	0.0007	0.0211	0.0044	0.0048	9.83 × $10^{-6}$
G10	0.0103	0.0142	0.0019	0.0001	0.0100	0.0080	0.0005	3.69 × $10^{-6}$
f(x)	3.2902	3.2839	3.2833	3.2789	16.5744	16.5625	16.5623	16.4885

## Data Availability

Not applicable.

## Abbreviations

The following abbreviations are used in this manuscript:

ACO	Ant Colony Optimization
BBO	Bio-geography-Based Optimization
BCHT	Boundary Constraint Handling Technique
CV	Constraint Violation
DE	Differential Evolution
IMO-CADE	Improved Multioperator-based Constrained Adaptive DE
OPA	Optimal Power Allocation
PSO	Particle Swarm Optimization
WSNs	Wireless Sensor Networks
